# Extreme risk protection order use in six US states: a descriptive study

**DOI:** 10.1186/s40621-025-00585-x

**Published:** 2025-06-03

**Authors:** Leslie M. Barnard, Marian E. Betz, Shannon Frattaroli, Christopher E. Knoepke, Annette Christy, Julia P. Schleimer, Veronica A. Pear, Megan McCarthy, Reena Kapoor, Michael A. Norko, Ali Rowhani-Rahbar, Wenjuan Ma, Garen J. Wintemute, Jeffrey W. Swanson, Michele M. Easter, April M. Zeoli

**Affiliations:** 1 Firearm Injury Prevention Initiative; University of Colorado Anschutz Medical Campus, Aurora, CO USA; 2 Colorado School of Public Health, Aurora, CO USA; 3 VA Eastern Colorado Geriatric Research Education and Clinical Center, Aurora, CO USA; 4 Johns Hopkins Bloomberg School of Public Health, Baltimore, MD USA; 5 College of Behavioral and Community Sciences, University of South Florida, Tampa, FL USA; 6 University of California Davis School of Medicine, Sacramento, CA USA; 7 Firearm Injury & Policy Research Program, University of Washington, Seattle, WA USA; 8 Department of Epidemiology, School of Public Health, University of Washington, Seattle, WA USA; 9 Yale University School of Medicine, New Haven, CT USA; 10 Center for Statistical Training and Consulting (CSTAT), Michigan State University, Lansing, MI USA; 11 Duke University School of Medicine, Durham, NC USA; 12 School of Public Health, University of Michigan, Ann Arbor, MI USA; 13 University of Michigan, Institute for Firearm Injury Prevention, Ann Arbor, MI USA

**Keywords:** Firearm violence, Extreme risk protection order

## Abstract

**Objectives:**

Extreme Risk Protection Orders (ERPOs) are civil court orders that temporarily prohibit firearm purchase and possession by someone (“respondent”) at imminent risk of harming themselves or others. Despite ERPOs being currently available in 21 states, DC, and U.S. V.I., little is known about the circumstances under which they are used across states.

**Methods:**

Using a standardized protocol, we abstracted ERPO petitions and associated court documents from 6 states to examine characteristics of respondents, documented risks of harm, and court outcomes. Included cases were filed through June 30, 2020, from 2013 (Connecticut) or from when the law went into effect (California: 2016; Colorado: 2020; Florida: 2018; Maryland: 2018; and Washington: 2016).

**Results:**

There were 6,634 ERPO petitions across included states. The median age of respondents was 40.0 years (SD: 16.4), and 10.8% were female. Almost half of petitions noted suicidal threats, plans, or ideation (43.9%) as the precipitating event, half noted interpersonal violence threats (50.8%), and one quarter (24.6%) noted threats to both self and others. Around one third (36.0%) noted unlawful or reckless firearm use. The majority of petitions (84.1%) indicated the respondent had current or recent access to a firearm. Most (77.5%) of the final orders (post-hearing) were granted. ERPO implementation varied across states, particularly with regard to how frequently they were used, for what type of threat, and by what type of petitioner.

**Conclusions:**

This study examined ERPO law implementation in 6 states, highlighting differences and similarities. This comparison allows for a more nuanced understanding of variation in ERPO use, which can inform ERPO implementation and future studies of ERPOs’ effectiveness.

## Introduction

Firearm injury is a leading cause of death in the United States [1]. During the late 20 th century, suicide (approximately 12 per 100,000 people) and homicide (approximately 9 per 100,000 people) rates remained relatively stable [1]. Between 2000 and 2022, there was a 32.5% increase in the suicide rate; the firearm suicide rate in 2022 and the homicide rate in 2021 were the highest on record in the 21 st century [1]. Death by suicide accounts for more than half of firearm deaths (56% in 2022), and firearms are the most common method of suicide (55% in 2022) [1]. Firearm suicide rates increased from 2021 to 2022 (7.9 to 8.1 per 100,000 population), and firearm homicide rates decreased slightly from 6.3 (per 100,000 population) in 2021 to 5.9 in 2022 [1, 2]. Reducing access to firearms when someone is illustrating risk of violence– including threatening suicide and/or violence directed at others– is a key approach to reducing firearm-involved injuries and deaths. One study found that about 1 in 5 suicide decedents who had been denied access to a firearm substituted another lethal method [3]. Removing the most lethal means of harm reduces the lethality of suicide attempts and the ramificiations of other-directed violence [4].

Extreme Risk Protection Orders (ERPOs), often referred to as “red flag laws,” seek to reduce firearm access for individuals at elevated risk of firearm-related violence. ERPO statutes establish a civil court process, including due process protections, that can temporarily prohibit firearm purchase and possession by individuals judged to be at imminent risk of harming themselves or others. ERPO-type laws have, as of May 2025, been enacted in 21 states, the District of Columbia, and U.S. Virgin Islands [5]. Under an ERPO, a “petitioner” files a petition with the court that includes evidence indicating that a “respondent” (the subject of the order) is at imminent risk of initiating violence. Petitioners are most commonly members of law enforcement, although family members, cohabitants, certain health and behavioral health professionals, educators, and others may also be authorized under individual state statutes. With a few exceptions, state processes prescribe that a judge reviews the petition in an emergency hearing (typically “*ex parte*,” without the respondent present). If an ERPO is granted, the respondent may not possess or obtain firearms while the order is in place and must relinquish any firearms already in their possession. At a subsequent hearing, typically 4–21 days later, as specified in statute, a judge determines whether to issue a “final” order extending the ERPO (in most states for up to one year). The respondent is notified of the hearing, given the opportunity to participate, and may be represented by a lawyer. In Colorado, the state will provide legal representation if the respondent requests it.

Firearm removal interventions have emerged in other countries including Canada (criminal procedure focusing on self harm, domestic and gender-based violence, 30 day removal with an option to extend up to 5 years) [6], Australia (intended to reduce firearm related-crime, results in cancellation of firearm license, not temporary) [7], and the United Kingdom (revocation or temporary restriction of firearms licenses) [8]. None of these policies mirror ERPOs in structure (most are crimial statutes with longer term removal periods) and there are limited studies examining their effectiveness. In the U.S. prior single-state or -county studies of ERPO laws have found mixed results and evidence of heterogenous implementation. For example, several studies from Connecticut and Indiana [9–11] have found that ERPO laws are associated with reductions in suicide at the state-level, and one study using individual-level data from Washington, Connecticut, California, and Maryland estimated that 13–23 ERPOs are needed to prevent one suicide [12]. One study performed in a single county in California did not find a reduction in firearm suicide [13]; however, this study has been characterized as premature in detecting population-level effects by Swanson and colleagues [14]. A Florida study found that firearm homicide mortality increased at a lower rate than would have been expected without ERPO but did not detect differences from expected firearm suicide mortality rates [15]. Prior literature has also examined ERPO use in individual states [16–19] in specific circumstances, such as mass shooting threats [20, 21] and cognitive impairment [22, 23], yet little is known about how different states with ERPO laws compare in terms of implementation.

Here, we examine patterns in ERPO use and circumstances across six states using a uniform data-gathering process in order to compare how states are using ERPOs. Findings can inform future efforts in policy development, implementation, and public education; support hypothesis generation around implementation and violence-related outcomes; and offer a standard format for reporting ERPO data in the literature.

## Methods

As previously described [21, 22, 24], we abstracted data from ERPO case files for petitions filed before July 1, 2020, starting from the time of law enactment (Table 1) in California, Colorado, Florida, Maryland, and Washington and starting in 2013 (several years after enactment) in Connecticut. (Connecticut’s law was enacted in 1999, but we included petitions starting from January 1, 2013, for comparability with other sites and because earlier Connecticut analyses have already been published [10]). In Florida, due to the high number of petitions, we coded a random sample of 50% of cases in counties with more than 10 ERPO cases; we coded 100% of cases in counties with fewer than 10 cases.

**Table 1:** Characteristics of ERPO laws in effect during abstraction period (through June 30, 2020), by state

Characteristic	CA	CO	CT	FL	MD	WA
Law name	Gun Violence Restraining Order (GVR0)	Extreme Risk Protection Order (ERPO)	Seizure of firearms and ammunition from person posing risk of imminent personal injury to self or others (Risk Warrant)	Risk Protection Order (RPO)	Extreme Risk Protective Order (ERPO)	Extreme Risk Protection Order (ERPO)
Date law took effect	1/1/2016	1/1/2020	6/29/1999	3/9/2018	10/1/2018	12/8/2016
Allowable petitioners	Law enforcement; family members; roommates; dating partners; people who share a child in common; and as of September 2020, employers, co-workers, employees and teachers at a secondary or postsecondary school the respondent has attended in the last 6 months	Law enforcement; family members; household members^a^	Law enforcement; state’s attorney	Law enforcement	Law enforcement; family members; licensed clinician^b^	Law enforcement; intimate partners^c^, family members^d^, or household members of the respondent.
Criteria for evaluating ERPO petitions	Person poses a significant danger in the near future of causing personal injury to self/others by having access to a firearm and when the removal of firearms is necessary to prevent injury; and less restrictive alternatives have been tried and found to be ineffective, or are inadequate or inappropriate for the circumstances	Person poses a significant risk to self/others by having a firearm in his/her custody/control or by possessing, purchasing, or receiving a firearm	Person who is at immediate risk of causing personal injury to themselves or another person and possesses firearms	Person poses a significant danger of harming himself or herself or others by possessing a firearm or ammunition	Person poses an immediate and present danger of causing personal injury to self or others by having firearms	Person poses a significant danger in the near future of causing personal injury to self or others by having custody or control of, purchasing, possessing, accessing, receiving, or attempting to purchase or receive firearms.
Changes to state laws during the study period	None	None	As of October 1, 2013, law permitted seizure of ammunition as well as firearms	None	None	In 2019, an amendment was made indicating that judges should consider whether the respondent had been convicted of “malicious harassment,” a category that includes hate-based behaviors
Ex parte temporary order (availability)	Yes (24/7 for law enforcement petitioners only^e^)	Yes (court hours)	Yes (judge’s warrant during court hours)	Yes (court hours)	Yes (24/7^e^)	Yes (court hours)
Length - Ex Parte temporary order	Up to 21 days	Up to 14 days	Up to 14 days	Up to 14 days	Up to 7 days	Up to 14 days
Length - full ERPO	Before September 2020, up to 1 year; after September 2020, up to 5 years	Up to 364 days	Up to 1 year	Up to 1 year	Up to 1 year	Up to 1 year
Requires that respondents be in possession of a firearm	No	No	Yes	No	No	No
Ex-parte process required before final order?	No	Yes	Judge’s warrant required for seizure of firearms/ammunition	No	No	No
Legal Counsel provided for respondents	No	Yes, court will appoint counsel at full order hearing	No	No	No	No

^a^ Includes people related by blood, marriage, or adoption, people with a child in common, people who regularly reside or regularly resided with the respondent in the last six months, domestic partners, parents, children, stepparents, stepchildren, grandparents, grandchildren, legal guardians, current or former unmarried couples

^b^ Includes physicians; psychologists; clinical social workers; licensed clinical professional counsellors; clinical nurse specialists in psychiatric and mental health nursing; psychiatric nurse practitioners; licensed clinical marriage or family therapists; and health officers or designees of health officers

^c^ Includes current or former spouse or domestic partners, current or former dating relationship (age 13 or older), persons who have a child in common (unless child was conceived through sexual assault)

^d^ Includes persons related by blood, marriage, domestic partnership, or adoption, persons who have a child in common, persons who reside or have resided with the respondent, persons who have a biological or legal parent-child relationship, including stepparents and step-children, grand-parents and grandchildren, and parent’s intimate partner and children• A person who is acting or has acted as the respondent’s legal guardian

^e^ 24/7 access is only limited to Law Enforcement in California while Maryland allows 24/7 access for everyone

Study staff obtained ERPO court files (e.g., ERPO petitions, affidavits, firearm ownership documents) for each state using a variety of public data access strategies and, where access was restricted, followed the required procedures to gain access. Abstracted case data were entered into a secure, centralized database. Abstractors received standardized training and double-coded with a lead coder until reaching an inter-rater reliability Kappa score of ≥ 0.80 [24].

Data elements abstracted included respondent demographics (age, gender, race, ethnicity); petitioner type (e.g., law enforcement officer [LEO], intimate partner, family member); risk behavior described during the precipitating event (e.g., threats or acts of harm against self or others, brandishing a weapon); documented firearm possession; and petition outcome (e.g., final hearing requested, orders granted or denied). ERPO court forms varied across states and sometimes across counties within states. Most data elements came from initial ERPO petitions, which generally included narratives written by the petitioner explaining respondents’ behavior as the factual basis for the petition. Petitions and other available court documents are not comprehensive records of behavior, histories, or mental health or substance use concerns, as petitioners may not have had access to information about respondents’ circumstances. Missing data elements were primarily due to their not being included in the original petition, and our decision not to code the absence of data as evidence that that element is not present (e.g., where a petition made no mention of a respondent’s substance use we coded substance use as “missing” as opposed to “no”; only those cases where no substance use was affirmatively stated were coded as “no”). Additionally, some counties in Colorado redacted certain identifiable data elements, such as dates of birth (or age), race, ethnicity, and medical history.

For this analysis, we sought to describe differences among ERPO laws and use across the six study states. We summarized distributions using frequencies and percentages for categorical variables and means (standard deviations), medians, and range for continuous variables. In order to avoid cells with small numbers where cases may be identifiable, cells with counts of 9 or fewer were pooled to arrive at a total larger than 10; we did not report on cells with counts of 10 or fewer. This approach was not used to address missing data, but rather to protect the identities of petitioners and respondents. We calculated average monthly ERPO petition rates per 100,000 population for each state (in Florida and California, we used the total number of petitions filed, not the number coded) by first dividing the number of petitions in the study period by the total state population (using 2020 Census estimates) [25]. To make rates comparable, we then divided those state rates by the total months each state contributed data in the study period (e.g., California was included from 1/1/2016-6/31/2020, contributing 54 months of data). All data cleaning and analyses were performed in R version 4.2.1 (Vienna, Austria) [26]. This study was approved by institutional review boards at each participating site.

## Results

While each state’s ERPO law is designed to prevent firearm violence, there are notable differences among state statutes (Table 1). Key differences include who is authorized to petition for an ERPO and under what circumstances. Law enforcement is always permitted to petition while four states also include combinations of family members, partners, co-workers, clinicians, and educators (Table 1). During the study period, only Maryland and California allowed petitions to be filed 24 h a day, 7 days a week, reducing barriers to access (however, this is only available to LEO in California). The six state laws also differ in terms of the enumerated criteria for the court to consider in making decisions to grant an ERPO. For example, California law specifies that an ERPO may be issued if “less restrictive alternatives have been tried and found to be ineffective, or are inadequate or inappropriate for the circumstances” (Table 1). Of note, only one of the six states (Connecticut) required a finding of probable cause that a respondent possesses a firearm as a criterion for issuing an ERPO, and that requirement changed after our study period (in 2022), permitting petitions for risk protection orders for respondents who did not possess firearms at the time of the petition. Another potentially important difference is that Colorado is the only state to provide court-appointed legal counsel to ERPO respondents at the hearing for the final order.

ERPO casefile analysis included 6,634 ERPO petitions filed through June 30, 2020, across six states (Table 2): 967 from California (14.6% of sample); 52 from Colorado (0.8%); 1,407 from Connecticut (21.2%); 1,347 from Maryland (20.3%), and 455 from Washington (6.9%). We also included 2,406 ERPO petitions from Florida (36.3% of study sample), sampled from 4,695 total petitions filed in that state. Maryland had the highest average monthly rate of ERPO petitions per 100,000 population (1.03), followed by Florida (0.79), Connecticut (0.42), Washington (0.19), Colorado (0.15), and California (0.07).

**Table 2:** Characteristics of ERPO respondents in petitions filed through June 30, 2020, by state (*N* = 6,634)

	Total		CA		CO		CT		FL		MD		WA	
	*N* = 6634		*n* = 967		*n* = 52		*n* = 1407		*n* = 2406		*n* = 1347		*n* = 455	
Rate of ERPO cases per month per 100,000 population	N/A		0.07		0.15		0.42		0.79		1.04		0.19	
**Respondent characteristics**														
	**n**	**%**	**n**	**%**	**n**	**%**	**n**	**%**	**n**	**%**	**n**	**%**	**n**	**%**
Female^b^ Male	718 5900	10.8 89.1	75 888	7.8 92.0	8 43	15.7 84.3	106 1301	7.5 92.5	302 2101	12.6 87.4	156 1184	11.6 88.4	71 383	15.6 84.4
Race/ethnicity^c^														
White	4343	65.5	591	61.1	36	69.2	680	48.3	1801	74.9	860	63.9	375	82.4
Black/African American	1032	15.6	96	9.9	NR^a^	-	35	2.5	428	17.8	443	32.9	28	6.2
American Indian/Alaska Native	15	0.2	NR^a^	-	NR^a^	-	NR^a^	-	NR^a^	-	NR^a^	-	NR^a^	-
Asian American	104	1.6	NR^a^	-	NR^a^	-	NR^a^	-	NR^a^	-	NR^a^	-	NR^a^	-
Native Hawaiian/Pacific Islander	10	0.2	NR^a^	-	NR^a^	-	NR^a^	-	NR^a^	-	NR^a^	-	NR^a^	-
Latino/Latina/Latinx/Hispanic	350	5.3	NR^a^	-	NR^a^	-	NR^a^	-	NR^a^	-	NR^a^	-	NR^a^	-
Other/Unknown	826	12.5	NR^a^	-	NR^a^	-	NR^a^	-	NR^a^	-	NR^a^	-	NR^a^	-
Veteran/Service Member	525	7.9	86	8.9	NR^a^	-	116	8.2	201	8.4	63	4.7	54	11.9
Documented criminal history	2345	35.4	323	33.4	31	59.6	65	4.6	1302	54.1	403	29.9	221	48.6
Evidence of current or recent access to firearms at the time of the petition^d^ Petition indicated how many firearms respondent possessed/could access before petition filed Mean # of firearms per respondent (range) Median # of firearms per respondent (SD, IQR)	5581 5084 4.5 2	84.1 76.7 1–700 13.6, 3	797 641 4.4 2	82.4 66.3 1–70 7.14, 4	39 39 6.4 3	75.0 5.0 1–59 12.3, 2	1397 1397 5.8 3	99.3 99.3 1–150 9.7, 5	1831 1831 3.9 2	76.1 76.1 1–700 18.3, 2	1127 1127 3.8 2	83.7 83.7 1–400 14.0, 2	390 390 3.7 2	85.7 85.7 1–37 4.3, 3
Does not possess or have access to a firearm	581	8.8	46	4.8	NR^a^	-	NR^a^	-	379	15.8	99	7.3	48	10.5
Possession unknown	472	7.1	124	12.8	NR^a^	-	NR^a^	-	196	8.2	121	9.0	17	3.7
**ERPO petitioner** ^e^														
Law enforcement	5950	89.7	944	97.6	26	50.0	1407	100.0	2404	99.9	765	56.8	404	88.8
Intimate partner	467	7.0	NR^a^	0.6	10	19.2	NA	NA	NA	NA	427	31.7	24	5.3
Other immediate family	134	2.0	NR^a^	-	NR^a^	-	NA	NA	NA	NA	100	7.4	20	4.4
Health professional	NR^a^	-	NA	-	NA	-	NA	NA	NA	NA	NR^a^	-	NR^a^	-
Other/unknown	74	1.1	NR^a^	-	NR^a^	-	NR^a^	-	NR^a^	-	48	3.6	NR^a^	-

^a^ Not reported if *N* < 10

^b^ Gender was known for 6621 cases; three petitions noted respondents as identifying as neither male nor female and are not presented here because they number fewer than 10. Other/unknown gender is also not included

^c^ Not reported if *N* < 10; Race/ethnicity categories are not mutually exclusive

^d^ Current or recent access at the time of the petition includes physical possession of a firearm, access to a firearm that is not their own, cases in which the respondent stored their gun with someone else, cases in which law enforcement took possession of the guns at the precipitating event, and cases in which law enforcement took possession of the guns in the weeks before the precipitating event. In Connecticut. this is the number of firearms that were seized, per court records

^e^ During the study period only law enforcement were authorized to file an ERPO petition in Connecticut and Florida

Among all petitions, respondents’ mean reported age was 42.1 (SD: 39.7) years, ranging from a mean of 38.4 years in Colorado to 47.6 years in Connecticut (Table 2). Most respondents were white (65.5%) and male (89.1%); 7.9% were reported to be current or former members of the military (Table 2).

Most petitions (84.1%) noted that the respondent had current or recent access to firearms at the time of the ERPO petition. Among states where firearm possession was not required for a petition to be filed (all states except for Connecticut), firearm access ranged from 75.0% in Colorado to 85.7% in Washington (Table 2). Most petitions where firearms were accessible to the respondent (76.7%) noted how many firearms the respondent possessed or could access. Among these, the median number of firearms per respondent was 2 (range: 1–700 IQR: 3; Table 2).

In states with multiple types of petitioners (California, Colorado, Maryland, and Washington), ERPO petitions were most often filed by law enforcement, ranging from 50.0% in Colorado to 97.6% in California (Table 2).

**Fig. 1:**
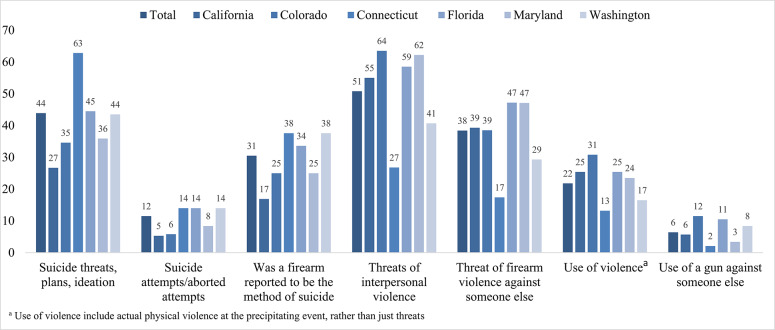
Documentation of precipitating event circumstances, ERPO petitions filed through June 30, 2020, by state (N=6,634; ≥1 allowed)

Nearly half of ERPO petitions noted suicidal threats, plans, ideation, or attempts/aborted attempts in the precipitating event (43.9%), ranging from 26.7% in California to 62.8% in Connecticut (Fig. 1). Approximately one third (30.5%) of the suicide threats, plans, ideations, or attempts/aborted attempts involved a firearm (as distinct from possession of or access to a firearm previously noted). Half of all ERPO petitions noted that interpersonal violence was threatened (regardless of method) in the precipitating event (50.8%) with substantial state variation (range: 26.8% in Connecticut to 63.5% in Colorado). Only 38.4% of these threats specifically described use of a gun (e.g. the respondent threatened to shoot themselves or others; range: 17.4% in Connecticut to 47.2% in Florida; Fig. 1). Almost 25% of petitions noted threats to both self and others by the respondent.

Most petitions (94.2%) began with an an *ex parte* petition. In the three states (Florida, Maryland, Colorado) that conduct *ex parte* hearings and where the study team had access to all filed petitions (as opposed to granted petitions only in California, Connecticut, and Washington), the combined mean percentage granted was 88.7%; in Florida, the percentage granted was over 98% (Table 3). Most (83.5%) *ex parte* petitions resulted in a final order hearing. Over three-fourths (77.5%) of final orders were granted (either “granted by a judge” or “stipulated to by the respondent”), with the highest percentage granted in Florida (94.2%) and the lowest in Connecticut (50.9%).

**Table 3:** Outcomes of ERPO petitions filed through June 30, 2020, by state (*N* = 6,634)

	Total		CA		CO		CT		FL		MD		WA	
	*N* = 6634		*n* = 967		*n* = 52		*n* = 1407		*n* = 2406		*n* = 1347		*n* = 455	
	**n**	**%**	**n**	**%**	**n**	**%**	**n**	**%**	**n**	**%**	**n**	**%**	**n**	**%**
Outcome of Ex parte hearing	4926		839 ^d^		50		NA^g^		2352		1234 ^a^		451^d^	
Granted ^b^	4508	91.5	839	100	30	60.0	NA	NA	2304	98.0	891	72.2	451	100
Dismissed ^b^	179	3.6	-	-	NR^h^	-	NA	NA	NR^a^	-	173	14.0	-	-
Denied ^b^	224	4.6	-	-	13	26.0	NA	NA	43	1.8	165	13.4	-	-
Unknown ^b^	15	0.3	-	-	NR^h^	-	NA	NA	NR^h^	-	NR^a^		-	-
Outcome of final hearing	5,538		694		27		1172		2,281		968		396	
Granted ^c, e^	4292	77.5	526	75.8	23	85.2	597	50.9	2,148	94.2	649	67.1	349	88.1
Dismissed ^c^	516	9.3	140	20.2	NR^h^	-	142	12.1	17	0.8	198	20.5	16	4.0
Denied ^c^	396	7.2	21	3.0	NR^h^	-	110	9.4	112	4.9	121	12.5	31	7.8
Unknown/other ^c, f^	334	6.0	NR^h^	-	NR^h^	-	323	27.6	NR^h^	-	NR^h^	-	NR^h^	-

^a^ To allow for comparison across states, we excluded Maryland cases that began as interim ERPO petitions and did not proceed to an ex parte or full order hearing. Interim ERPO petitions are filed outside of normal court hours and are decided by a judicial commissioner

^b^ Denominator for % is Ex parte requested

^c^ Denominator for % is where there was a final order hearing

^d^ CA and WA only received data for cases in which an emergency or ex parte order was granted

^e^Granted is combined categories of “granted by a judge” and “stipulated to by the respondent.”

^f^ In Connecticut, the court checks “other” rather than “dismissed” where the firearms were transferred to a third party or the respondent agrees to have them destroyed

^g^ During an ex parte hearing in CT, law enforcement make a verbal report to the court and if the ex parte ERPO is granted, it generates paper records. If it isn’t, there’s no record of it therefore, granting and denial rates are not reportable

^h^ Not Reported (NR), cells with counts of 10 or fewer

## Discussion

This is the first study to use a standardized data collection approach to describe ERPO utilization and court outcomes across multiple states. While the goal of each state law is to prevent firearm violence (directed at self or others) by temporarily restricting firearm access among those whose behaviors indicate high risk for violence, state laws vary in how they seek to accomplish this. This study highlights the differences and similarities across states in how ERPOs are being used and allows for a more nuanced understanding of the possible effects of varied policies and their implementation.

ERPO use varied across the six states, with the highest average monthly rate per population in Maryland. Differences in state laws may partially explain this finding as Maryland provides for 24/7 court access (California also does, but only to law enforcement petitioners). Rates may also be lower in California and Washington where we only had access to cases where an emergency or ex parte order was granted. The standard for California’s law requires that less restrictive means are inappropriate, inadequate, or ineffective in order to proceed with an ERPO, which may also lead to lower rates. Connecticut’s longstanding law may have gained greater public awareness than in other states and may explain that state’s higher use rate while Colorado’s law was implemented in 2020 which may explain its lower use rate. Summarizing state rates is complicated by unequal ERPO implementation within states, as use tends to be concentrated in particular counties and jurisdictions; average state-wide rates thus mask variation in ERPO rates across localities in the same state.

Our findings on the proportion of cases involving a respondent with access to firearms carry implications for state laws that require an individual to already have access to firearms in order to be subject to an ERPO. About 15% of the cases we evaluated would not be granted in states requiring firearms to be possessed when the ERPO is issued; these respondents would therefore remain eligible to purchase a firearm despite behaving in a way that demonstrates imminent dangerousness. Specifically, ERPO laws aim to intervene when dangerous behaviors are escalating to prevent firearm violence from occurring, and as such, ERPOs can be used to both remove firearms and prevent firearm acquisition during times of crisis. One study from California found that 2.5% of ERPOs in that state (called gun violence restraining orders) were issued while the respondent was in the waiting period to purchase a firearm [18]. Additionally, we found that 38.4% of EPRO threats described use of a gun; this may indicate that the combination of threats and access to firearms is sufficient for judges to grant an ERPO.

Connecticut is the only state in our study with a large majority of petitions sought in response to suicide risk. This continues the trend from 1999 to 2013 in Connecticut, previously described [10]. Notably, the age-adjusted suicide rate (10.6 in CT vs. 14.2 in the U.S. per 100,000) and age-adjusted firearm suicide rate (3.4 in CT vs. 7.6 in the U.S. per 100,000) in Connecticut were lower than the national average in 2022.

Civilian petitioners were rare in California and Washington compared with Colorado and Maryland (only these four study states allowed civilian petitioners during the study period). Approximately 45% of petitions in Colorado and Maryland were filed by non-law enforcement petitioners, compared to less than 12% of petitions in California and Washington. Differences may be due, in part, to variation in knowledge about ERPOs and how to file a petition. For example, a survey of California adults found that 66% of respondents had not heard of the state’s ERPO law in 2020 [27]. Beyond knowledge of the law, prior research points to other barriers and facilitators to petitioning. For example, despite the high percentage of civilian petitioners in Maryland, very few were physicians. In prior studies, Maryland physicians cited time as the main barrier to petitioning and suggested several strategies to make ERPOs a viable intervention for practicing clinicians, including increasing access to education and legal consultation, allowing clinicians to testify remotely, and providing a trained coordinator who could file petitions and attend court hearings [28, 29]. Similarly, in Washington state, civilian petitioners cited prior legal experience and specialized advocates who helped them with the petition and court processes as facilitators to filing a petition [30]. County and municipal policies may influence who files petitions. In Colorado, the propotion of petitions filed by law enfocement is lower in “Second Amendment Sanctuary” jurisdictions– possibly due to policies implicitly discouraging the use of ERPOs and/or compensatory filing by other allowable petitioners [31]. Access to the court may also be a factor in whether civilians decide to petition; for example, Maryland allows for all petitions to be heard 24 h a day, 7 days a week.

Racial equity in policy application is an important area for ongoing study [32]. Our analysis revealed that some states had higher percentages of ERPO respondents identified as Black compared to the state’s population (e.g., 6.5% of California’s population [33] is Black, and 9.9% of California ERPO respondents were Black) and others lower (e.g., 12.9% of Connecticut’s population is Black, and 2.5% of respondents were Black). Racial equity in policy application, however, is not as straightforward as equating the percentage of a particular population to ERPO respondents within that population. The burden of firearm injury across racialized groups is not equal [1], and ERPO laws are designed to prevent firearm injury risk. We might therefore expect proportions of use to more closely mirror firearm injury risk levels than the proportion of the population represented by any particular group. The likelihood of becoming an ERPO respondent is also informed by firearm access, risk factors for violence, access to other resources to promote safety, willingness of family to utilize ERPO, and whether risk is made known to law enforcement. These factors, other social and structural determinants of crime, violence, and mental health outcomes [34] (including suicide), and biased institutional responses may produce inequities in use or effectiveness of these laws. If ERPOs are differentially effective, then strides must be made to improve both the use and effectiveness of the intervention.

Examination of ERPO petitions provides insight with regard to racial equity and ERPO use. It is also important to consider public opinion and interest in ERPOs as a violence prevention tool. In a survey of Californians, there was overall high willingness to petition for an ERPO when a family member was exhibiting signs of potential future violence [35], but Black adults had the lowest levels of support (64.1% vs. 72.9% or higher among all other racial/ethnic groups), citing lower knowledge of ERPOs and more distrust of the system, although the small number of Black respondents (N = x) could produce unstable estimates [35].

This work lends itself to additional research. First, the differences in implementation practices within and between states require further exploration. In addition, identifying and pursuing strategies to address the factors affecting racial and ethnic differences in reported willingness to use ERPO is important to informing equitable implementation– this should include culturally-sensitive trainings, education campaigns, and implementation protocols [32, 36]. Additional research should further evaluate all aspects of equity in the application of ERPOs across states, including issues related to reasons for filing, the underlying population at risk, effectiveness, and the intersection of race with other ERPO characteristics such as intimate partner violence and suicide threats. Previous research has proposed that people of color may be less likely to call police or petition the courts in these cases [35] or that firearm permittees in CT are predominantly White, so the heavy skew of ERPO respondents mostly represents who owns guns in the state [37]. Combined with our findings, this additional research can help identify opportunities for policy development, implementation improvements, and public education efforts.

As with most research conducted using court documents, one limitation of this study is that the data in the documents themselves were not collected for research purposes. Therefore, petitioners within and across jurisdictions in different states may have conceptualized risk behaviors differently, and the petitions themselves (which vary across jurisdictions) may be more conducive to including more details for certain variables in some states relative to others. Additionally, petitioners in different states may have varied approaches to including certain information in the petitions, resulting in differences in information captured through the petitions we reviewed that may not reflect respondent differences. Finally, this analysis was at the case level (with most cases being considered at both the ex parte and final stages), not the individual level, so a person who was a respondent to more than one ERPO was counted more than once. Despite these limitations, the scope of this data collection and analysis effort provides new opportunities to understand and compare ERPO uptake in six heterogeneous states.

## Conclusion

This study examined ERPO laws and their implementation in 6 states, highlighting differences and similarities. Across states, we observed variability in petitioners, respondent demographics, reasons for petitions, and outcomes, including the proportion of ERPOs granted. In addition, this analysis provides a holistic picture of how the ERPO process is unfolding in the six states examined. This inter-state comparison allows for a more nuanced understanding of ERPO policies and their implementation than previous single-state studies and may inform practice and generate hypotheses about ERPOs’ effectiveness.

## Data Availability

The dataset supporting the conclusions of this article will become available in the institute for firearm injury prevention data repository, part of icpsr, (https://www.openicpsr.org/openicpsr/facts-open) upon publication of related manuscripts as allowed by participating states.

## Abbreviation

ERPO: Extreme risk protection order
